# Atmospheric sulfur is recycled to the crystalline continental crust during supercontinent formation

**DOI:** 10.1038/s41467-018-06691-3

**Published:** 2018-10-22

**Authors:** Crystal LaFlamme, Marco L. Fiorentini, Mark D. Lindsay, Thi Hao Bui

**Affiliations:** 1 Département de géologie et de génie géologique, Université Laval, Pavillon Adrien-Pouliot 1065, av. de la Médecine, Bureau 4309, Québec, QC G1V 0A6 Canada; 2 Centre for Exploration Targeting, ARC Centre of Excellence for Core to Crust Fluid Systems (CCFS), School of Earth Sciences, University of Western Australia, 35 Stirling Highway, Crawley, WA 6009 Australia; 3 Department of Earth and Planetary Sciences and GEOTOP, McGill University, 3450 University Street, Montreal, QC H3A 0E8 Canada

## Abstract

The sulfur cycle across the lithosphere and the role of this volatile element in the metasomatism of the mantle at ancient cratonic boundaries are poorly constrained. We address these knowledge gaps by tracking the journey of sulfur in the assembly of a Proterozoic supercontinent using mass independent isotope fractionation (MIF-S) as an indelible tracer. MIF-S is a signature that was imparted to supracrustal sulfur reservoirs before the ~2.4 Ga Great Oxidation Event. The spatial representation of multiple sulfur isotope data indicates that successive Proterozoic granitoid suites preserve Δ^33^S up to +0.8‰ in areas adjacent to Archean cratons. These results indicate that suturing of cratons began with devolatilisation of slab-derived sediments deep in the lithosphere. This process transferred atmospheric sulfur to a mantle source reservoir, which was tapped intermittently for over 300 million years of magmatism. Our work tracks pathways and storage of sulfur in the lithosphere at craton margins.

## Abstract (web summary)

The long-term evolution of the sulfur budget in the lithosphere is poorly constrained. Here, using mass independent isotope fractionation as an indelible tracer, the authors track the pathway of sulfur from the Earth’s surface to punctuated episodes of granitoid magmatism during collisional orogenesis.

## Introduction

Numerous studies have recently advocated that in the Archean Eon, the margins of lithospheric blocks, played a first-order control on the ascent and focusing of mantle plumes^1,2^. This process led to the emplacement of large volumes of magmas which are host to some of the greatest accumulations of sulfide-rich precious and base metals on Earth^3,4^. As the Archean Eon waned, a significant secular evolution in the nature of many of the Earth’s reservoirs occurred^5^; these changes are preserved in the rock record of cratonic margins^6^. However, whereas voluminous crustal growth through granitoid magmatism is known to coincide with the formation of a supercontinent^7^, the transfer mechanism of volatile elements such as sulfur during the assembly of lithospheric blocks remains poorly understood.

Early initiation of a supercontinent cycle amalgamates cratons through a series of subduction zones^8^, above which juvenile crust forms in magmatic arcs^9^. It is commonly assumed that the radiogenic isotope composition of the mantle-extracted juvenile crust should be identical to that of the depleted mantle at the time when crust was formed^10^. However, the radiogenic isotope signature of the depleted mantle is commonly absent in these rocks. To unravel this paradox, it has recently been hypothesized that the subduction-driven incorporation of sediments derived from the erosion of continental crust to the mantle may significantly alter its depleted isotopic signature^11^. To test this hypothesis, we investigate the flux of Archean surficial sedimentary rocks, a reservoir that preserves a unique and indelible sulfur isotope signature, through the lithosphere. As sulfur can form a volatile compound, it is mobile in and sensitive to fluids, thus tracking a pathway that is traceable even to the deepest parts of the lithosphere^12,13^.

Sulfur carries two isotopic signatures that have been fundamental in understanding terrestrial processes including biogeochemical cycles and the redox evolution of the oceans and atmosphere through time^14^. Variations in δ^34^S reflect mass-dependent fractionation (MDF-S) during chemical exchange between sulfur-bearing reservoirs as sulfur progresses through its planetary cycle. Complementary to MDF-S, the phenomenon of mass-independent fractionation of sulfur (MIF-S) results in fractionation of ^33^S and ^36^S away from the mass-dependent fractionation relationship with ^34^S (quantified as positive and negative Δ^33^S and Δ^36^S). The MIF-S signature (Δ^33^S and Δ^36^S ≠ 0‰) was generated through the photodissociation of S-bearing gases by short wavelength ultraviolet rays in the Archean oxygen-poor atmosphere prior to the Great Oxygenation Event (GOE) at ~2.45−2.33 Ga^15–17^. Both reduced (carried as +Δ^33^S) and oxic (carried as −Δ^33^S and/or +Δ^33^S^18^) forms of sulfur were deposited in the oceanic water column, most commonly in iron- and/or carbon-rich sediments in marginal basins that were proximal to sources of atmospheric gases^19^.

The juvenile crystalline crust and the underlying depleted mantle that has not experienced metasomatism are thought to be MIF-S absent^12,20,21^; however, recent studies have identified MIF-S in localized magmatic environments that source the deep lithosphere, including diamond sulfide inclusions^22–24^, basaltic plumes^25,26^, and igneous provinces^27,28^. To elaborate on these findings and understand the process related to the transfer of sulfur through the lithosphere, we track the fate of Archean MIF-S-bearing sediments through the first post-GOE supercontinent cycle of Nuna/Columbia. By spatially mapping the MIF-S signature of a Proterozoic orogen, it is possible to monitor through time the ancient sulfur flux across lithospheric reservoirs. Our dataset indicates that the Nuna/Columbia supercontinent cycle commenced with a significant amount of sediment devolatilization in the lithosphere which resulted in the transfer of Archean sulfur molecules (originally contained in marine sediments) to Proterozoic mantle-derived granitoid melts during punctuated pulses of magmatism for over 300 million years.

## Results

### Proterozoic craton margin setting

**Fig. 1:**
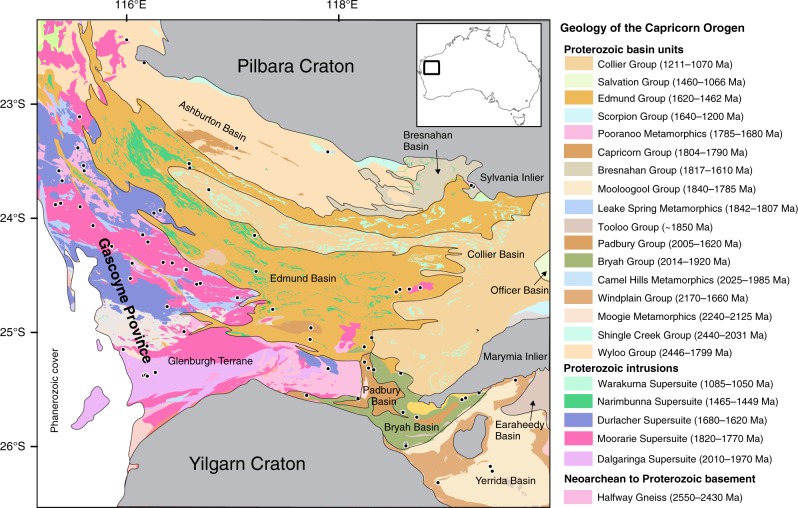
Geological map of the Capricorn Orogen with sample locations. Black dots are locations of samples analyzed for multiple sulfur isotopes to form the interpolated Δ^33^S isotope model. Ages of formal groups from the Geoscience Australia’s Australian Stratigraphic Units Database

Our natural laboratory to trace volatile movement across tectonic boundaries is the Proterozoic Capricorn Orogen of Western Australia which developed between two sulfur-rich and metal-endowed Archean crustal blocks: the Yilgarn and Pilbara cratons (Fig. 1). The Capricorn Orogen of Western Australia formed during the amalgamation of cratons worldwide into the 2.0–1.8 Ga supercontinent Nuna/Columbia^29,30^. The oldest crustal fragment in the Capricorn Orogen is the Neoarchean-Paleoproterozoic Glenburgh Terrane which is composed of 2.55–2.43 Ga granitoid gneisses. The ~1000 km-long, ~500 km-wide belt underwent at least two collisional events known as the 2.22–2.15 Ga Ophthalmia Orogeny and the 2.01–1.95 Ga Glenburgh Orogeny, when the Glenburgh Terrane was accreted to the Pilbara and Yilgarn cratons, respectively^31^.

The Glenburgh Orogeny is expressed by 2.0 Ga arc granitoid magmatism of the Dalgaringa Supersuite. Subsequently, the Capricorn Orogen experienced over a billion years of magmatism (including intrusion of the post-orogenic 1.8 Ga Moorarie and 1.65 Ga Durlacher supersuites), intracontinental reworking, and basin development^32^. Overall, the Capricorn Orogen is composed of variably deformed and metamorphosed Paleoproterozoic to Neoproterozoic magmatic and supracrustal rocks with overlying sedimentary basins^32^. More details pertaining to the geology of the Pilbara and Yilgarn cratons as well as the Capricorn Orogen are presented in the Supplementary Note 1.

The aim of this study is to spatially constrain the nature and rate of MIF-S recycling from the nearby Archean Yilgarn and Pilbara cratons to the post-GOE lithologies of the Capricorn Orogen. We investigate the multiple sulfur isotope signature recorded in sulfides (i.e., the mineral phases that contain the reduced sulfur ion S^2−^) which in a post-GOE terrain reflects the input of both oxidized and reduced sulfur-bearing reservoirs^18^. Multiple sulfur isotope analyses were performed on 74 Proterozoic samples from across the Capricorn Orogen and include magmatic (*n* = 54), sedimentary (*n* = 14), and hydrothermal (*n* = 6) lithologies. Samples were crushed and powdered. Sulfides were extracted from the samples for isotopic analysis completed by fluorination coupled with isotope ratio mass spectrometry. Repeat analyses on IAEA-S1, IAEA-S2, and IAEA-S3 determine that uncertainty (2 SD) on δ^34^S, Δ^33^S, and Δ^36^S is better than ±0.15‰, ±0.02‰, and ±0.3‰, respectively.

**Fig. 2:**
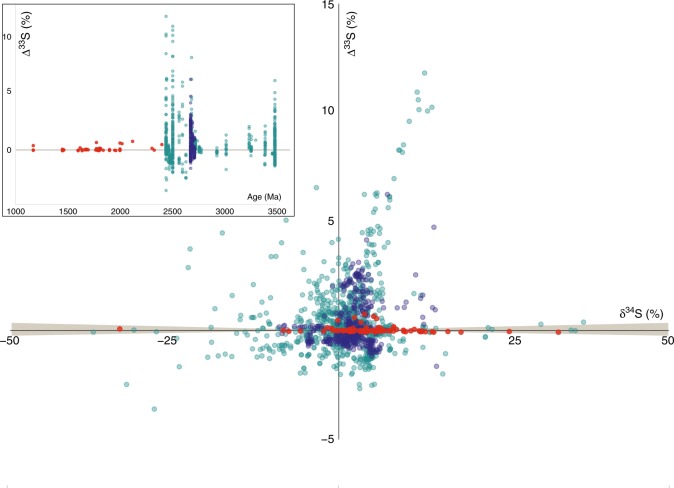
Multiple sulfur isotope results. Results from the Capricorn Orogen (bright red; *n* = 78) overlain on a compilation of published multiple sulfur isotope data from the Pilbara (green; *n* = 840) and Yilgarn (blue; *n* = 381) cratons. Beige area represents limit of MDF-S^34^. Data are cited in the Supplementary Information. Error bars are in every instance smaller than the symbol. Inset of same data plotted as Δ^33^S versus age with beige line representing the extent of MDF-S

Results from this study are overlain on a database of published δ^34^S–Δ^33^S values from the Pilbara and Yilgarn cratons (Fig. 2). Values of δ^34^S are dominantly positive and range from −33.1 to +40.0‰, and values of Δ^33^S are primarily positive and range from −0.07 to +0.80‰. Values of Δ^36^S are dominantly negative and range from −0.87 to +1.14‰. Lithological and geochronological details pertaining to each of the samples and their multiple sulfur isotope results are presented in Supplementary Data 1. Further details pertaining to sample selection, analytical methods, and equations for sulfur isotope calculation are provided in the Supplementary Note 1.

### MIF-S in the Proterozoic crystalline continental crust

Anomalous Δ^33^S values >0.1‰ occur in 14 samples (out of 74). These samples include 2.0–1.65 Ga syn- to post-orogenic granitoids (*n* = 8; Δ^33^S = +0.1 to +0.6‰) in which disseminated pyrite is the host of sulfur, a pelitic lens within a syn-orogenic granitoid (Δ^33^S = +0.75‰), and Proterozoic sulfide mineralization near the boundary between the Pilbara Craton and Capricorn Orogen (*n* = 4; Δ^33^S = +0.11 to +0.65‰). Finally, a single sample of ca. 2.3 Ga metasandstone yields Δ^33^S of +0.14‰. However, the +Δ^33^S preserved in this specific sample may be derived from direct interaction with the atmosphere. In fact, small Δ^33^S values of up to +0.34‰ in 2.45–2.09 Ga sedimentary rocks have been documented elsewhere and interpreted to reflect a post-GOE waning period for photodissociation in the atmosphere^15^.

The magnitude of the Δ^33^S and Δ^36^S values observed here is too large to be the result of MDF-S processes alone. It has been demonstrated that in localized environments, shifts in Δ^33^S can be controlled by a number of MDF-S mechanisms involving bacteria and organic molecules at low temperatures (<~200 °C^16^). In such systems, dispersions to Δ^33^S values of up to 0 ± 0.2‰ can occur^17^. However, this phenomenon is possible only at fractionated values of δ^34^S equal to less than –15‰ or greater than +15‰^33,34^, which are not commonly produced in the Archean Eon^35^ nor reflected in the MIF-S-bearing subsample set of this study (δ^34^S = –1.6 to +8.4‰). In addition, experimental work has documented a magnetic isotope effect during thermochemical sulfate reduction to impart ∆^33^S anomalies^36,37^; however, experimental simulations of these processes are not able to reproduce the observed deviations in Δ^36^S that are preserved by a subset of samples from this study. Rather, large dispersions in Δ^33^S and Δ^36^S are significant in the supracrustal rock record deposited before ca. 2.4 Ga and are controlled by the production, transfer, and preservation of MIF-S signals in an O_2_-poor early Earth atmosphere^16^. Further, the subset of samples with a MIF-S component yields a Δ^33^S-Δ^36^S array of −1.2, which is consistent with the Archean Δ^33^S–Δ^36^S array^16,34^.

**Fig. 3:**
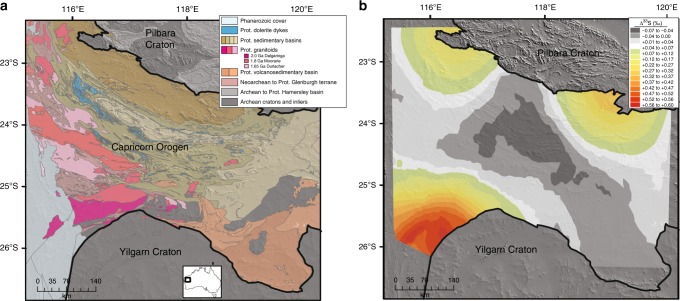
Interpolated model of MIF-S within Capricorn Orogen. **a** Location and geology of the Proterozoic Capricorn Orogen between the Archean Pilbara and Yilgarn cratons in Western Australia. **b** Interpolated model of Δ^33^S for Proterozoic samples of the Capricorn Orogen by ordinary kriging to show that MIF-S occurs in Proterozoic rocks located along the margins of the Archean Pilbara and Yilgarn cratons

We present the dataset spatially in order to illustrate where the anomalously positive Δ^33^S signature is preferentially concentrated across the Capricorn Orogen (Fig. 3). We present an interpolated model using ordinary kriging, a geostatistical method that allows the mean to have an unknown value while assuming it is constant. Interpolation of the multiple sulfur isotope data (all 74 samples) was performed to display the spatial variation of Δ^33^S and facilitate visual assessment of measured values with the mapped geology. Whereas an interpolated model of radiogenic isotope data is used to contour ages or model ages of the crust, the interpolated model presented here is used to visually highlight the proximity of the anomalous Proterozoic lithologies along the margins of the Pilbara and Yilgarn cratons. This spatial association lends insight into the tectonic mechanisms responsible for sulfur (re)-cycling during assembly and stitching of a supercontinent.

## Discussion

We interpret the preferential siting of anomalously positive Δ^33^S signatures along the margins of the Archean cratons as evidence that MIF-S can be imparted to the Proterozoic crystalline rock record by tectonically driven crust formation processes. By broadening our study spatially and temporally across the entire orogen, multiple sulfur isotope results indicate that sulfur recycling occurs over a very protracted time span, as it is associated with at least three magmatic pulses and over 300 million years of crust formation. Prior to laying out the arguments that support our new hypotheses, it is important to emphasize that the physical transport of detrital MIF-S-bearing sulfides upon erosion of Archean sedimentary platforms is unlikely, because chemical weathering and dissolution of detrital pyrite occurs within short transport distances in the post-GOE surficial environment^38^. This is in agreement with the observation that weathering of Archean sulfides is not a likely mechanism to transport and disperse a MIF-S signature^39^ across different surficial environments.

**Fig. 4:**
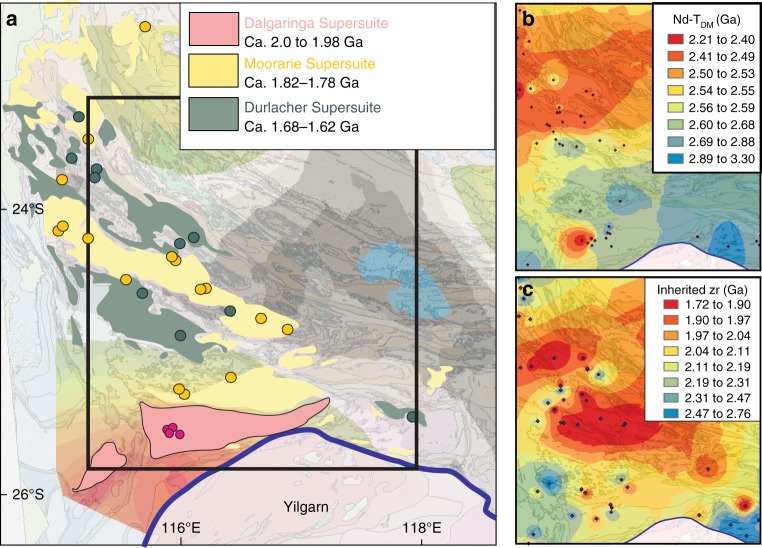
Time slice comparison of MIF-S and conventional radiogenic isotope tracers as seen through the Proterozoic granitoid rock record. **a** Location of supersuites and samples analyzed for multiple sulfur isotopes. Transparent background of Fig. 3a, b. **b** Interpolated whole rock Nd depleted mantle model ages. **c** Interpolated average inherited U-Pb in zircon ages. Data^46^ in **b** and **c** interpolated using an inverse distance weighted interpolation

To demonstrate how sulfur isotope tracers can lend insight into crust formation mechanisms related to recycling, transfer, mobility, and focusing of sulfur along lithospheric pathways, we investigate the nature of magmatism in specific Proterozoic time slices pertinent to the supercontinent cycle (see Fig. 4a). The earliest of these magmatic events, exposed in the south-west margin of the orogen as the Dalgaringa Supersuite, formed as a ca. 2.0 Ga I-type magmatic arc during ocean closure and subsequent collision between the composite Pilbara Craton–Glenburgh Terrane with the Yilgarn Craton^40^. The 2.0 Ga Dalgaringa Supersuite contains ubiquitous MIF-S (Δ^33^S = +0.3 to +0.8‰; average = +0.57 ± 0.15‰ (1*σ*)) in granitoids and intervening pelitic lenses, as well as in sulfides associated with orogenic Au^27^, demonstrating that magmatism was at least partially derived from an Archean supracrustal reservoir. Evidence of 2.0 Ga arc magmatism that preserves the anomalous MIF-S signature provides the opportunity to investigate the contribution of the sediments in the formation of crust extracted from the depleted mantle.

The presence of a MIF-S-bearing Proterozoic arc at the margin of the Capricorn Orogen may reflect assimilation of the anomalous signature into the juvenile granitic melts from the surrounding crust^28^. In fact, the sulfur isotope signature of granite magmas generally reflects the signature of the sulfide-rich sedimentary country rocks that are assimilated upon emplacement^41^. However, Archean sedimentary rocks that may contain anomalous sulfur values have not been identified within the Glenburgh Terrane. In addition, assimilation of any concealed Archean sulfide-rich sedimentary rocks at depth would have likely increased the sulfur contents of the MIF-S-bearing granitoids. However, this is implausible, as the analyzed granitoids display average crustal sulfur contents (100s of ppm S^42,43^). Furthermore, the 2.55–2.43 Ga Halfway Gneiss intruded by the Dalgaringa Supersuite is largely composed of metamorphosed I-type magmatic rocks, which generally yield less than 50 ppm S^27^. Therefore, it is unlikely that the observed anomalous sulfur signatures in the Proterozoic arc magmas were inherited through crustal contamination.

The anomalously positive Δ^33^S signatures in the Proterozoic granitoids are more likely inherited from their sources, reflecting either (1) metasomatism of the mantle wedge during subduction of Archean sediments at the incipient stages of the supercontinent cycle^8^ and/or (2) local anatexis of the MIF-S-bearing lower crust in the overriding plate. To elucidate the two scenarios, we compare our results with the available radiogenic isotope datasets. Radiogenic isotope tracers such as Sm-Nd and Lu-Hf have long been used to fingerprint the presence of any older crustal material previously extracted from the mantle^44^. As the mantle becomes progressively depleted, it is assumed that the radiogenic isotope composition of the newly formed juvenile crust is identical to that of the depleted mantle^10^; however, this signature is underrepresented in the ancient rock record^11^. Whereas Sm-Nd and Lu-Hf are specifically sensitive to recycling, assimilation, and differentiation of the ancient crystalline continental crust, the elements Sm, Nd, Lu, and Hf are not readily partitioned into aqueous fluids at conditions typical of slab dehydration (up to 4 GPa/800 °C)^45^ and, therefore are not specifically sensitive to sediment-driven mantle metasomatism.

The integration of the newly developed MIF-S tracer with conventional radiogenic isotopes may elucidate the presently incomplete picture. The preservation of MIF-S in the 2.0 Ga Dalgaringa arc granitoids is in agreement with evidence from other isotopic tracers, such as inherited U-Pb and Lu-Hf in zircon^46^, and whole rock Sm-Nd^40^. Collectively, these data indicate that the Dalgaringa Supersuite incorporated a significant proportion of reworked crust. As arc magmatism leads to the formation of juvenile melts from differentiation of mantle-derived mafic magmas^9^, it is argued that the MIF-S signature preserved in the 2.0 Ga Dalgaringa arc granitoids uniquely fingerprints a mantle previously metasomatized by the devolatilization of MIF-S-bearing Archean sediments. To test this hypothesis, it is necessary to investigate whether the Pilbara and Yilgarn cratons may be the ultimate source of the MIF-S-bearing rocks recorded in the Proterozoic rocks of the orogen.

The margin of the Pilbara Craton proximal to the Capricorn Orogen is composed of the ca. 2.63–2.45 Ga volcanosedimentary Hamersley Group for which shale, banded iron formation, and dolomite yield Δ^33^S values of −3‰ to +8‰ (*n* = 545) and a positive δ^34^S–Δ^33^S array (see Fig. 2 for source of data). Even if the sulfur budget of the Yilgarn Craton is less well studied, it is known that magmatic and hydrothermal sulfides hosted in greenstone supracrustal belts yield Δ^33^S values of −1‰ to +3‰ (see Fig. 2 for source of data). A deep seismic reflection survey images the suturing of the Yilgarn and Pilbara cratons with the Glenburgh Terrane, from which tectonic models describe subduction of the two aforementioned cratons beneath the crustal substrate to the Capricorn Orogen^32^. Therefore, data are consistent with the hypothesis that devolatilization of Archean anomalous MIF-S-bearing sediments from the Yilgarn and Pilbara cratons have contributed to metasomatism of the mantle source of the juvenile Proterozoic granitoid magmas of the Capricorn Orogen.

As the accretion of the Nuna/Columbia progressed, two temporally distinct peraluminous granitoid supersuites (ca. 1.80 Ga Moorarie and ca. 1.65 Ga Durlacher supersuites) were emplaced in an intracontinental environment^46^ coincident with the supercontinent-wide post-orogenic granite ‘bloom’^47^. The genesis of such intracontinental peraluminous magmatic events is interpreted to be related to anatexis of the lower crust and mixing with melts derived from partial melting of lithospheric mantle following asthenospheric upwelling^47^_._ The Moorarie and Durlacher supersuites are exposed along an array that is orthogonal to the orogenic belt^32^. Granitoids that were emplaced during these two magmatic events only locally preserve a MIF-S signature at the margins of the Capricorn Orogen (Δ^33^S = +0.1 to +0.2‰), but dominantly yield no MIF-S in the central part of the orogen (Δ^33^S = 0‰). Following localized metasomatism of the mantle by Archean sediments during subduction, the MIF-S was stored in the mantle and recycled during two pulses of post-orogenic magmatism.

To test this hypothesis, we compare our multiple sulfur isotope results with available conventional radiogenic isotopes collected across the orogen^46^, including whole rock Sm-Nd and average inherited U-Pb in zircon. These datasets reveal that the most evolved Proterozoic magmas are located proximal to the Archean cratons (Fig. 4b, c). Our dataset is in accordance with this interpretation, whereby the MIF-S tracer uniquely fingerprints the recycling and incorporation of sulfur from Archean sedimentary rocks into mantle-derived magmas emplaced proximal to the Archean cratons. It is proposed that the MIF-S-signature is transferred firstly to 2.0 Ga arc magmatism, and then locally recycled twice more during the post-orogenic granite ‘bloom’ at 1.80 and 1.65 Ga.

**Fig. 5:**
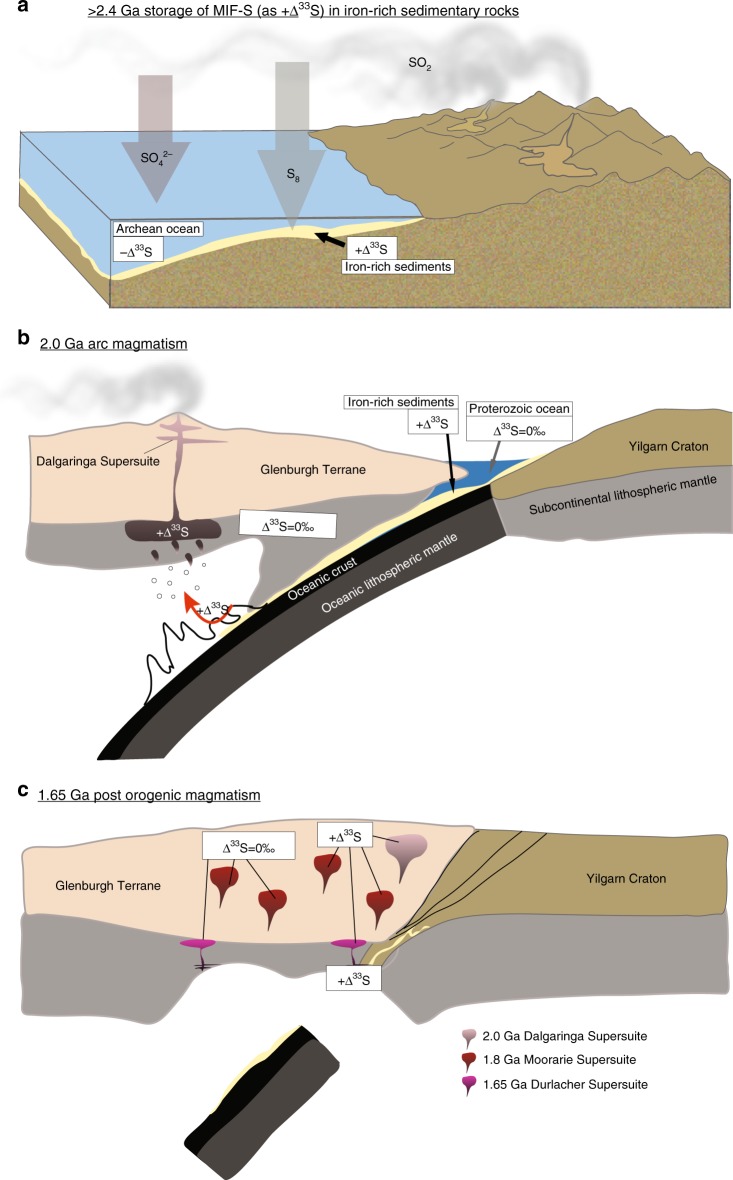
Model for transfer of surficial sulfur from surface to crystalline crust through time splices. **a** Deposition of MIF-S-bearing aerosols into the water column some time prior to 2.4 Ga. The positive +Δ^33^S signature is preserved in iron-rich sedimentary rocks outboard of the Yilgarn Craton. **b** As the Yilgarn Craton collides with (and subducts beneath) the Glenburgh Terrane during ca. 2.0 Ga ocean closure^32^, MIF-S-bearing sedimentary rocks devolatilize and volatiles are transferred (captured as +Δ^33^S) to mantle-derived arc melts. **c** Post-orogenic magmatism generates MIF-S-bearing plutons close to the margin of the Yilgarn Craton, locally recycling the +Δ^33^S signature from the deep lithospheric MIF-S-bearing reservoir to the ca. 1.8 Ga Moorarie Supersuite (time splice not shown) and the ca. 1.65 Ga Durlacher Supersuite

The incomplete picture provided by conventional radiogenic isotopes can be enhanced by the proposed MIF-S tracer, which is able to track sediment devolatilization and image the cryptic network of volatile transfer pathways among different lithospheric reservoirs. These observations support the conclusion that the assembly of Nuna/Columbia began with significant recycling of sulfur (and likely also other volatiles) from subducted Archean sediments to punctuated magmatic events spanning 300 million years. One possible geodynamic scenario that is consistent with all available datasets is presented in Fig. 5.

Throughout Earth’s evolution, the generation^7^ and/or preservation^6^ of significant new granitoid crust has been associated with punctuated episodes of magmatism during the supercontinent cycle. However, juvenile Proterozoic continental crust is lithologically and chemically distinct to that formed prior to 3.0 Ga^5^. The nature and evolution of Archean to Proterozoic crustal formation processes remains a contentious debate in which studies focus primarily on the chemical and radiogenic isotopic signature of melts^6,7,48,49^. However, the Archean granitoid rock record has not been reported to contain MIF-S. Therefore, the local preservation of anomalous MIF-S in the Proterozoic granitoid rock record may suggest a secular change in the processes related to crust generation across the critical Archean-Proterozoic transition period.

## Methods

### Sample information

Details including lithologic descriptions, units assigned, geochronology, and location information pertaining to samples analyzed are located in the Supplementary Table. Further details regarding samples provided by the GSWA are available at Geological Survey of Western Australia, Compilation of Geochronology Information, 2016 Update, Digital Data Product^50^ which is available to the public at https://geoview.dmp.wa.gov.au/GeoViews/?Viewer=GeoVIEW.

Geoview requires the installation of Silverlight™. Sample locations are shown in Fig. 1.

Because sulfur is commonly associated with hydrothermal mineralization, a small portion of the samples reflect sulfides from these environments. These include one sample of sulfides from each of the following deposits: Prairie Downs, Wolf, Paulsens, Glenburgh, Mt Olympus, Degrussa, Abra. In each of these deposits, sulfides are either hosted in Proterozoic-age rocks (e.g., Wolf, Mt Olympus), have been dated to be Proterozoic in age (Paulsens, Prairie Downs), or both (Degrussa, Glenburgh). See Supplementary data for ages and citations.

### Multiple sulfur isotope analysis

Multiple sulfur isotope analysis was completed at the Stable Isotope Laboratory of the Department of Earth and Planetary Sciences at McGill University, Montreal, Canada. For whole rock samples without visible sulfur-bearing phases, sulfur was chemically extracted from 5–15 g powders to form Ag_2_S by Cr reduction and follow previously defined methods^51^. Depending on the lithology, ~0–15 mg of Ag_2_S was yielded from the extraction process. Approximately 2 mg of Ag_2_S was converted into SF_6_ by fluorination at ~225 °C. The SF_6_ was purified cryogenically and underwent gas chromatography. The gas was then injected into a ThermoFinnigan MAT 253 dual-inlet gas source mass spectrometer to analyze *m/z* 127, 128, 129, and 131. Sulfur isotope data were normalized to repeated measurements of international reference material IAEA-S-1 (δ^33^S_V-CDT_ = –0.061‰; δ^34^S_V-CDT_ = –0.3‰; δ^36^S_V-CDT_ = –1.27‰). An additional 32 whole rock samples yielded <0.5 mg of Ag_2_S (the minimum for analysis) and could not be analyzed. For samples with 0.5–1.0 mg of yielded Ag_2_S, a microanalytical method utilized a microvolume and modified resistor capacities^52^. Location, lithological, and geochronological details of the samples as well as multiple sulfur isotope data and detailed methods are presented in the Supplementary Note 1.

The precision and accuracy of the bulk fluorination system is evaluated by repeat analyses that return uncertainty (2 SD) on δ^34^S, Δ^33^S, and Δ^36^S values as better than ±0.15‰, ±0.02‰, and ±0.3‰, respectively. Sulfur isotopic ratios are expressed on the Vienna-Canyon Diablo Troilite (V-CDT) scale^53^. The δ^34^S values are calculated by the following equation:

$${\mathrm{\delta}}^{34}{\mathrm{S}} = \left[ {\frac{{\left( {\,^{34}{\mathrm{S}}/\,^{32}{\mathrm{S}}} \right)_{{\mathrm{sample}}} - \left( {\,^{34}{\mathrm{S}}/\,^{32}{\mathrm{S}}} \right)_{{\mathrm{reference}}}}}{{\left( {\,^{34}{\mathrm{S}}/\,^{32}{\mathrm{S}}} \right)_{{\mathrm{reference}}}}}} \right] \times 1000.$$

The Δ^33^S and Δ^36^S values are calculated as follows:

$$\Delta ^{3x}{\mathrm{S}} = \delta ^{3x}{\mathrm{S}}_{\mathrm{measured}} - \left[ {\left( {\frac{{\delta \,^{34}{\mathrm{S}}_{\mathrm{measured}}}}{{1000}} + 1} \right)^{3x\lambda } - 1} \right] \times 1000,$$

to approximate high temperature conditions^16^, utilizing a value for the slope of the mass-dependent fractionation line equal to 0.515 and 1.9 for δ^33^S and δ^36^S, respectively^54,55^.

### Model interpolation

The isotope model was interpolated using ordinary kriging, a geostatistical method that allows the assumed mean to have an unknown value while assuming it is constant^56,57^ and chosen for interpolation to reflect the fact that the source of sulfur is assumed to be locally consistent. Kriging was chosen to interpolate data in ArcGIS™, as the source of sulfur is assumed to be locally consistent. In addition, it is a stochastic interpolation scheme, and can make statistically valid predictions from the input^56^. A maximum of five and a minimum of two sample values were used to calculate the isotopic value at a given location. A low maximum value was assigned to compensate for the sparse sample coverage, especially in the east and north-east regions. Trend analysis was performed and did not detect any systematic global data trends. Model error was calculated by comparing the interpolated isotopic values with the measured values at each sample point. As expected, the largest errors are observed at sample locations near the edge of the dataset or isolated locations but were within an acceptable error range^2^.

## Electronic supplementary material

Supplementary Information

Description of Additional Supplementary Files

Supplementary Data 1

## Data Availability

The data that support Fig. 2 are cited in the Supplementary Information and are publically available at http://www.cet.edu.au/research-projects/special-projects/gssid-global-sedimentary-sulfur-isotope-database. Data generated and analyzed in this study are included in Supplementary Data 1.
