# Supplementary material for: Atmospheric sulfur is recycled to the crystalline continental crust during supercontinent formation
Source: Nat Commun. 2018 Oct 22;9:4380. doi: 10.1038/s41467-018-06691-3 (PMC6197212; doi:10.1038/s41467-018-06691-3)
Supplement: Supplementary file 1 — Supplementary Information [file 41467_2018_6691_MOESM1_ESM.docx]

**Atmospheric sulfur is recycled to the crystalline continental crust during supercontinent formation**

LaFlamme, Crystal^*^, Fiorentini, Marco L., Lindsay, Mark D., Bui, Hao Thi

**SUPPLEMENTARY NOTE 1**

**Regional Geology**

Pilbara Craton

The Pilbara Craton is dominantly composed of granite-greenstone basement rocks older than ca. 2.77 Ga^1^. Within the Capricorn Orogen, the Pilbara Craton is exposed in several inliers, the Sylvania, Rocklea, Milli Milli and Wyloo Inliers, along the northern margin of the orogen. The craton is overlain by mafic volcanic, volcaniclastic and sedimentary rocks of the Fortescue and Hamersley Basins which formed by 2.78 Ga crustal extension and volcanic plateau volcanism (Fortescue Group), through passive margin settings (2.63–2.45 Ga Hamersley Group)^1^. Crustal extension began with mafic and felsic volcanic plateau volcanism (2.78-2.63 Ga Fortescue Group) followed by siliciclastic sedimentation and continued with the deposition of banded iron formation, shale and carbonate in a passive margin setting (2.63-2.45 Ga Hamersley Group).

Yilgarn Craton

The Yilgarn Carton is an extensive region of Archean crust dominated by granite-greenstone belts^2^. The craton has been subdivided into several tectonic units, including the Narryer, Youanmi, and Yamarna Terranes and the Eastern Goldfields Superterrane, many of which have margins that intersect the Capricorn Orogen. These units generally contain north-south-striking greenstone belts separated by granite and gneiss. Extensive tectonometamorphic reworking and magmatic intrusion occurred at the margins of these terranes prior to, and during, the 1.82–1.77 Ga Capricorn Orogeny.

Capricorn Orogen

The geological packages of the Capricorn Orogen have been previously been described in detail^3^ and are briefly detailed below. The Gascoyne Province is host to the oldest exposed lithologies in the orogen, known as the Glenburgh Terrane which is made up of the 2.55–2.43 Ga granitic Halfway Gneiss interpreted to be reworked 2.73–2.60 Ga Archean gneisses^4,5^. The Halfway Gneiss includes rock types of interlayered leucocratic granitic gneiss and foliated leucocratic metagranite, mesocratic granitic gneiss, augen gneiss, pegmatite-banded tonalitic and granodioritic gneiss, pale-grey granitic gneiss, gneissic to foliated porphyritic metagranodiorite, foliated metagranite, and metapegmatite^5^. The Glenburgh Terrane is also host to ca. 2.2 Ga metasedimentary rocks, the Moogie Metamorphics, which were deposited in a foreland basin during accretion of the Pilbara and Glenburgh Terranes during the 2.22–2.15 Ga Ophthalmia Orogeny^3^. No Archean sediments have been identified in the Glenburgh Terrane.

Emplacement of arc-related granitic rocks of the 2.00–1.97 Ga Dalgaringa Supersuite along the south-west margin of the composite Pilbara Craton–Glenburgh Terrane took place during the onset of collision with the Yilgarn Craton^6,7,8,9^. Continent-continent collision resulted in the amalgamation of the West Australian Craton^6^.

Associated with, and following this tectonic event, siliciclastic and mafic volcano-sedimentary rocks were deposited in the Yerrida, Earaheedy, Bryah and Padbury basins^10^ to the east. These basins formed between ca. 2.20 and 1.80 Ga, and record periods of sedimentation and volcanism associated with rifting, accretion, and passive margin tectonism. The 2.26–2.17 Ga Yerrida Basin is composed of siliciclastic, stromatolitic carbonate, and evaporatic rocks at the base which are derived from the Yilgarn Craton, and are overlain by ca. 1.76 Ga ferruginous clastic mafic volcanic rocks. The 2.02–1.85 Ga Earaheedy Basin is comprised of shallow-marine turbiditic sandstones, siltstone and carbonate rocks that have been deformed into an asymmetric east-plunging regional syncline. The 2.05–1.99 Ga Bryah Basin is comprised of mafic to ultramafic intrusive and volcaniclastic rocks that were formed, or deposited, in a rift environment. These units are overlain by high-energy, siliciclastic metasedimentary rocks of the 2.01–1.80 Ga Padbury Basin that form rift-fill successions. To the west, this is synchronous with the deposition of the ca. 1.84 Ga Leake Springs Metamorphics. At the top of the succession extensive banded iron formations and ferruginous sedimentary rocks are present that were deposited sometime between ca. 1.80 and 1.60 Ga.

The Capricorn Orogen is also host to felsic magmatic rocks of the 1.82–1.77 Ga Moorarie and 1.68–1.62 Ga Durlacher Supersuites, that were generated during intracratonic reworking events of the Capricorn and Mangaroon Orogenies, respectively^3,4,11,12^. The Capricorn Orogeny also resulted in deposition of siliciclastic sedimentary rocks into the Ashburton Basin, along the southern margin of the Pilbara Craton. The Mangaroon Orogeny also saw deposition of the Pooranoo Metamorphics in the west. Following the Mangaroon Orogeny, sedimentation took place in the 1.68–1.07 Ga Edmund and Collier Basins which consist of 4–10 km of siliciclastic and carbonate rocks that were deposited under fluviatile to deep-marine conditions^13^. The Edmund Basin forms four major sequences of dominantly silisticlastic sedimentary rocks including siltstone, mudstone, sandstone, and conglomerate with minor carbonate rocks. The basin formed in an intracontinental extensional setting and was deposited between 1.68 and 1.45 Ga in series of half grabens. The 1.17–1.07 Ga Collier Basin forms two major depositional sequences that are composed of shale, mudstone, siltstone, sandstone, and minor dolostone. Both these basins were intruded by numerous dolerite sills between 1.51–1.07 Ga. The youngest rocks in the region, which overlie the Capricorn Orogen, are a succession of mixed carbonate-clastic-evaporite rocks^14^ that form part of the Neoproterozoic to Cambrian Officer Basin.

**Geological, chemical and isotopic investigations of granitoid suites**

The following section is an in depth investigation of previously compiled geological, geochronological, chemical and isotopic studies of the granitoid crust forming the Capricorn Orogen. A compilation of the isotopic framework (U-Pb, Nd, Hf, O) of the four igneous events forming the granitic crust of the Capricorn Orogen has been published^15^.

2.55–2.43 Ga Halfway Gneiss

The Halfway Gneiss forms the oldest identified portion of the Capricorn Orogen, and combined with the younger Dalgaringa Supersuite (below) comprises what is known as the Glenburgh Terrane. The geology, geochemistry, geochronology, and Hf isotopes of the Halfway Gneiss have previously been reported^16^. The Halfway Gneiss is composed of tonalite to monzogranite and contains cm- to m-scale granite veins that define a gneissic fabric^6^. Importantly, supracrustal sequences have not been observed within the Halfway Gneiss. The Halfway Gneiss contains abundant inherited zircon ranging in age from ca. 2.6 Ga to ca. 3.56 Ga. Hf isotopic studies of magmatic and inherited zircon demonstrate an array to ca. 3.7 Ga, indicating that the Halfway Gneiss was formed by crustal melting and remelting of older protoliths without significant addition of juvenile mantle-derived material. The sulfur content of the Halfway Gneiss has been investigated^17^, and yielded sulfur contents below the extraction threshold (<50 ppm).

2.01–1.97 Ga Dalgaringa Supersuite

In addition to the isotopic dataset^15^, a geological, chemical and Nd isotope study of the Dalgaringa Supersuite have previously been published^7^. The supersuite comprises sheets, dykes and veins of 2.01–1.97 Ga foliated and gneissic I-type tonalite, granodiorite, quartz diorite and monzogranite. The Dalgaringa Supersuite has been metamorphosed up to granulite-facies conditions during the Glenburgh Orogeny^6^. It contains localised, rare and isolated 5-10 m-wide strips and lenses of pelitic diatexite with maximum depositional ages of ~2.12 Ga. Rocks of the Dalgaringa Supersuite do not intrude the adjacent Yilgarn Craton and documented geological relationships indicate that the Dalgaringa Supersuite formed at a Palaeoproterozoic plate margin during subduction. Whole-rock major, trace and rare earth element compositions are similar to those in Phanerozoic Andean-type batholiths; however, initial εNd values are slightly more evolved (+1.0 to −6.0)^7,15^. Hf isotopes are also evolved and are dominantly negative, ranging from ~-11 to +2 with one outlier at +6^15^.

The Dalgaringa Supersuite is interpreted to have formed in a magmatic arc due to northward subduction prior to collision of the Yilgarn Craton and Glenburgh Terrane^7^. However, based on relatively low εNd and εHf values and elevated SiO_2_ contents, the supersuite is interpreted to contain a significantly higher proportion of older crust than most Phanerozoic Andean-type batholiths^7^. The evolved isotopic component of the Nd and Hf arrays is interpreted to reflect mixing, melting and assimilation of the Halfway Gneiss of the Glenburgh Terrane with juvenile basaltic melts^15^. The whole-rock major and trace element compositions also indicate that the magmatic rocks were generated from a chemically heterogeneous source in the middle to deep crust^7,15^. The sulfur isotope composition of the Dalgaringa Supersuite and associated sulfide mineralisation in a small orogenic gold deposit has locally been investigated^17^. Results demonstrate that all samples analysed yield Δ^33^S = +0.22 – +0.77‰.

1.82–1.77 Ga Moorarie Supersuite

The geology and chemistry of the Moorarie Supersuite has previously been described^4^. Overall, the Moorarie Supersuite forms a series of granodiorite and monzogranite plutons which are relatively silicic (61–77 wt.% SiO_2,_ average = 70 wt.% SiO_2_), peraluminous, and have calc-alkalic major and trace element compositions. Compositionally, the Moorarie Supersuite varies across the orogenic belt from north to south indicating either variable source melts and/or assimilation of different layers of crust during emplacement Rocks of basic and intermediate composition are scattered rafts throughout the plutons.

Inherited zircons yield a restricted range of U–Pb dates between ca. 2.28 and 2.12 Ga^15^. Initial εNd values range from -2 to -8^4^ and initial εHf values range from +5 to -14, and are dominantly negative^15^. The strong vertical arrays in initial εNd and initial εHf space, and the presence of radiogenic mafic material, suggests that the Mooraire Supersuite was generated by the interaction and variable mixing between a source similar to Depleted Mantle melts and evolved crust^15^.

The range of initial εHf and δ^18^O magmatic zircon compositions define at least three end-member isotopic component that are spatially constrained from north to south^15^. (1) radiogenic initial εHf compositions and light δ^18^O values, similar to that of the depleted mantle; (2) variably evolved initial εHf compositions and heavy δ^18^O values characteristic of sedimentary and upper crustal rocks; and, (3) variably evolved initial εHf compositions with relatively light to moderate δ^18^O values which might represent a deep- or mid-crustal component.

1.68–1.62 Ga Durlacher Supersuite

The Durlacher Supersuite forms relatively silicic (59–76 wt.% SiO_2,_average = 70 wt.% SiO_2_), peraluminous grandiorite to syenogranite plutons that have calc-alkalic major and trace element compositions. The geology and geochronology of the Durlacher Supersuite are previously described^12^.

Inherited zircons yield a range of U–Pb dates between ca. 2.68 and 1.70 Ga^15^. Isotopic studies demonstrate initial εNd that ranges from -5 to -9 and initial εHf that ranges from 0 to -14^15^. The distinct lack of elevated Lu/Hf ratios, and the absence of any associated mafic material, suggests that these granitic rocks may have been generated in a setting without the involvement of significant depleted mantle melts.

The primary source of the Durlacher Supersuite is interpreted to be melting and recycling of mid-crustal granitic rocks with compositions the Moorarie Supersuite^15^. Similarly to the Moorarie Supersuite, the Durlacher Supersuite contains a more evolved array in the south than the north.

**Supplementary References**

1-Hickman, H.A. & Van Kranendonk, M.J. Early Earth evolution: evidence from the 3.5-1.8 Ga history of the Pilbara region of Western Australia. *Episodes* **35**, 283-297 (2012).

2-Cassidy, K.F., et al. A revised geological framework for the Yilgarn Craton, Western Australia. *Geological Survey of Western Australia Record* **2006/8**, 8 pp (2006).

3-Johnson, S.P., et al. Crustal architecture of the Capricorn Orogen, Western Australia and associated metallogeny, *Aust. J. Earth Sci.* **60**, 681-705 (2013).

4-Sheppard, S., Bodorkos, S., Johnson, S.P., Wingate, M.T.D. & Kirkland, C.L. The Paleoproterozoic Capricorn Orogeny: intracontinental reworking not continent–continent collision. *Geological Survey of Western Australia Report* **108**, 33 pp (2010).

5-Johnson, S.P., Sheppard, S., Wingate, M.T.D., Kirkland, C.L. & Belousova, E.A. Temporal and hafnium isotopic evolution of the Glenburgh Terrane basement: an exotic crustal fragment in the Capricorn Orogen. *Geological Survey of Western Australia Report* **110**, 27 pp (2011).

6-Occhipinti, S.A., Sheppard, S., Passchier C., Tyler, I. M. & Nelson, D.R. Palaeoproterozoic crustal accretion and collision in the southern Capricorn Orogen: the Glenburgh Orogeny. *Precambrian Res*. **128**, 237-255 (2004).

7-Sheppard S., Occhipinti S.A. & Tyler, I.M. A 2005–1970 Ma Andean-type batholith in the southern Gascoyne Complex, Western Australia. *Precambrian Res.* **128**, 257-277 (2004).

8-Johnson, S.P., et al. Two collisions, two sutures: punctuated pre-1950 Ma assembly of the West Australian Craton during the Ophthalmian and Glenburgh Orogenies. *Precambrian Res.* **189**, 239-262 (2011).

9-Martin D.M., & Morris, P. A. Tectonic setting and regional implications of ca 2.2 Ga mafic magmatism in the southern Hamersley Province, Western Australia. *Aust. J. Earth Sci.* **57**, 911-931 (2010).

10-Pirajno, F. & Occhipinti, S.A. Three Palaeoproterozoic basins – Yerrida, Bryah and Padbury – Capricorn Orogen, Western Australia. *Aust. J. Earth Sci.* **47**, 675-688 (2001).

11-Evans D.A.D., et al. Revised geochronology of magmatism in the western Capricorn Orogen at 1805–1785 Ma: Diachroneity of the Pilbara–Yilgarn collision. *Aust. J. Earth Sci.* **50**, 853-864 (2003).

12-Sheppard, S., Occhipinti, S.A. & Nelson, D.R. Intracontinental reworking in the Capricorn Orogen, Western Australia: the 1680–1620 Ma Mangaroon Orogeny. *Aust. J. Earth Sci.* **52**, 443-460 (2005).

13-Martin, D.M. & Thorne, A. M. 2004. Tectonic setting and basin evolution of the Bangemall Supergroup in the northwestern Capricorn Orogen. *Precambrian Res.* **128**, 385-409 (2004).

14-Grey, K., et al. Lithostratigraphic nomenclature of the Officer Basin and correlative parts of the Paterson Orogen Western Australia. *Geological Survey of Western Australia Report* **93**, 95 pp (2005).

15-Johnson, S.P., et al. An isotopic perspective on growth and differentiation of Proterozoic crust: from subduction magmatism to cratonization. *Lithos* **268**, 76-86 (2017).

16-Johnson, S.P. Sheppard, S., Wingate, M.T.D., Kirkland, C.L. & Belousova, E.A. Temporal and hafnium isotopic evolution of the Glenburgh Terrane basement: an exotic crustal fragment in the Capricorn Orogen. *Geological Survey of Western Australia Report* **110** (2011).

17-Selvaraja, V., Fiorentini, M.L., LaFlamme, C., Wing_,_ B.A. & Bui, T.H. Anomalous isotope signatures trace sulfur pathways in magmatic arcs. *Geol.* (2017).

**Citations for Figure 2**

1. A. Bekker *et al.,* Atmospheric sulfur in Archean komatiite-hosted nickel deposits. *Sci.* 326, 1086-1089 (2009).

2. T. R. Bontognali *et al.,* Sulfur isotopes of organic matter preserved in 3.45-billion-year-old stromatolites reveal microbial metabolism. *Proc. Natl. Acad. Sci. USA* 109, 15146-15151 (2012).

3. M. Chen *et al.,* Multiple sulfur isotope analyses support a magmatic model for the volcanogenic massive sulfide deposits of the Teutonic Bore volcanic complex, Yilgarn Craton, Western Australia. *Econ. Geol.* 110, 1411-1423 (2015).

4. S. D. Domagal-Goldman, J. F. Kasting, D. T. Johnston, J. Farquhar, Organic haze, glaciations and multiple sulfur isotopes in the Mid-Archean Era. *Earth Planet. Sci. Lett.* 269, 29-40 (2008).

5. J. Farquhar *et al.,* Isotopic evidence for Mesoarchaean anoxia and changing atmospheric sulphur chemistry. *Nature* 449, 706-709 (2007).

6. J. Farquhar, H. Bao, M. Thiemens, Atmospheric influence of Earth’s earliest sulfur cycle. *Sci.* 28, 756-758 (2000).

7. M. L. Fiorentini, S. W. Beresford, W. E. Stone, E. Deloule, Evidence of water degassing during emplacement and crystallization of 2.7 Ga komatiites from the Agnew-Wiluna greenstone belt, Western Australia. *Contrib. Mineral. Petrol.* 164, 143-155 (2012).

8. S. D. Golding *et al.,* Earliest seafloor hydrothermal systems on Earth- Comparison with modern analogues. In S.D. Golding and M. Glikson (Ed.), *Earliest life on earth: Habitats, environments and methods of detection*. New York, U.S.A.: Springer-Verlag Dordrecht. pp. 15-49 (2011).

9. D. D. Gregory *et al.,* Trace element content of pyrite from the Kapai Slate, St. Ives Gold District, Western Australia, *Econ. Geol.* 111, 1297-1320 (2016).

10. G. Hu, D. Rumble, P. Wang, An ultraviolet laser microprobe for the in sity analysis of multisulfur isotopes and its use in measuring Archean sulfur isotope mass-independent anomalies. *Geochim. Cosmochim. Acta* 67, 3101-3118 (2003).

11. A. J. Kaufman *et al.,* Late Archean biospheric oxygenation and atmospheric evolution. *Sci.* 317, 1900-1903 (2007).

12. É. Muller, P. Philippot, C. Rollion-Bard, P. Cartigny, Multiple sulfur-isotope signatures in Archean sulfates and their implications for the chemistry and dynamics of the early atmosphere: *Proc. Natl. Acad. Sci. USA* 113, 7432-7437 (2016).

13. S. J. Mojzsis, C. D. Coath, J. P. Greenwood, K. D. McKeegan, T. M. Harrison, Mass-independent isotope effects in Archean (2.5-3.8 Ga) sedimentary sulfides determined by ion microprobe analysis. *Geochim. Cosmochim. Acta* 67, 1635-1658 (2003).

14. H. Ohmoto, Y. Watanabe, H. Ikemi, S. R. Poulson, B. E. Taylor, Sulphur isotope evidence for an oxic Archaean atmosphere. *Nature* 442, 908-911 (2006).

15. M. A. Partridge, S. D. Golding, K. A. Baublys, E. Young, Pyrite paragenesis and multiple sulfur isotope distributions in late Archean and early Proterozoic Hamersley Basin sediments. *Earth Planet. Sci. Lett.* 272, 41-49 (2008).

16. P. Philippot *et al.,* Early Archaean microorganisms preferred elemental sulfur, not sulfate. *Sci.* 317, 1534-1537 (2007).

17. Y. Shen, J. Farquhar, A. Masterson, A. J. Kaufman, R. Buick, Evaluating the role of microbial sulfate reduction in the early Archean using quadruple isotope systematics. *Earth Planet. Sci. Lett.* 279, 383-391 (2009).

18. E. D. Swanner *et al.,* Geochemistry of pyrite from diamictites of the Boolgeeda Iron Formation, Western Australia with implications for the GOE and Paleoproterozoic ice ages. *Chem. Geol*. 362, 131-142 (2013).

19. C. Thomazo *et al.,* Biological activity and the Earth’s surface evolution: insights from carbon, sulfur, nitrogen and iron stable isotopes in the rock record. *C.R. Palevol.* 8, 665-678 (2009).

20.Y. Ueno, S. Ono, D. Rumble, S. Maruyama, Quadruple sulfur isotope analysis of ca. 3.5 Ga Dresser Formation: New evidence for microbial sulfate reduction in the early Archean. *Geochim. Cosmochim. Acta* 72, 5675-5691 (2008).

21. D. Wacey, N. Noffke, J. Cliff, M. E. Barley, J. Farquhar, Micro-scale quadruple sulfur isotope analysis of pyrite from the 3480 Ma Dresser Formation: new insights into sulfur cycling on the early Earth. *Precambrian Res.* 258, 24-35 (2015).

22. K. H. Williford, M. J. Van Kranendonk, T. Ushikubo, R. Kozdon, J. Valley, Constraining atmospheric oxygen and seawater sulfate concentrations during Paleoproterozoic glaciation: in situ sulfur three isotope microanalysis of pyrite from the Turee Creek Group, Western Australia. *Geochim. Cosmochim. Acta* 75, 5686-5705 (2011).
