# Supplementary material for: Atmospheric sulfur is recycled to the crystalline continental crust during supercontinent formation
Source: Nat Commun. 2018 Oct 22;9:4380. doi: 10.1038/s41467-018-06691-3 (PMC6197212; doi:10.1038/s41467-018-06691-3)
Supplement: Supplementary file 2 — Description of Additional Supplementary Files [file 41467_2018_6691_MOESM2_ESM.pdf]

Description of Additional Supplementary Files

File Name: Supplementary Data 1
Description: Isotopic Map Data
